# Demographic histories shape population genomics of the common coral grouper (*Plectropomus leopardus*)

**DOI:** 10.1111/eva.13450

**Published:** 2022-08-05

**Authors:** Samuel D. Payet, Morgan S. Pratchett, Pablo Saenz‐Agudelo, Michael L. Berumen, Joseph D. DiBattista, Hugo B. Harrison

**Affiliations:** ^1^ Australian Research Council Centre of Excellence for Coral Reef Studies James Cook University Townsville Queensland Australia; ^2^ Instituto de Ciencias Ambientales y Evolutivas Universidad Austral de Chile Valdivia Chile; ^3^ Division of Biological and Environmental Science and Engineering, Red Sea Research Center King Abdullah University of Science and Technology Thuwal Saudi Arabia; ^4^ Australian Museum Research Institute, Australian Museum Sydney New South Wales Australia; ^5^ Australian Institute of Marine Science Townsville Queensland Australia

**Keywords:** fisheries management, molecular evolution, population genetics – empirical

## Abstract

Many coral reef fishes display remarkable genetic and phenotypic variation across their geographic ranges. Understanding how historical and contemporary processes have shaped these patterns remains a focal question in evolutionary biology since they reveal how diversity is generated and how it may respond to future environmental change. Here, we compare the population genomics and demographic histories of a commercially and ecologically important coral reef fish, the common coral grouper (*Plectropomus leopardus* [Lacépède 1802]), across two adjoining regions (the Great Barrier Reef; GBR, and the Coral Sea, Australia) spanning approximately 14 degrees of latitude and 9 degrees of longitude. We analysed 4548 single nucleotide polymorphism (SNP) markers across 11 sites and show that genetic connectivity between regions is low, despite their relative proximity (~100 km) and an absence of any obvious geographic barrier. Inferred demographic histories using 10,479 markers suggest that the Coral Sea population was founded by a small number of GBR individuals and that divergence occurred ~190 kya under a model of isolation with asymmetric migration. We detected population expansions in both regions, but estimates of contemporary effective population sizes were approximately 50% smaller in Coral Sea sites, which also had lower genetic diversity. Our results suggest that *P. leopardus* in the Coral Sea have experienced a long period of isolation that precedes the recent glacial period (~10–120 kya) and may be vulnerable to localized disturbances due to their relative reliance on local larval replenishment. While it is difficult to determine the underlying events that led to the divergence of the Coral Sea and GBR lineages, we show that even geographically proximate populations of a widely dispersed coral reef fish can have vastly different evolutionary histories.

## 1 INTRODUCTION

Coral reef fishes have broad distributions, which are attributed to their capacity to disperse as pelagic larvae (Jones et al., 2002) and an apparent rarity of strong isolating barriers in the ocean (Bowen et al., 2013). Consequently, populations establish in a range of geographic locations, leading to unique evolutionary interactions with their surrounding environment. The product of these interactions is evident across the seascape because they manifest as phenotypic and genetic variations at local scales (e.g., Choat & Robertson, 2002; Gaither et al., 2015; Pinheiro et al., 2017; Rocha et al., 2007). However, the processes that contribute to these patterns and the temporal scale of their emergence remain focal questions in evolutionary biology.

Since most coral spawners and are relatively site‐associated as adults, population connectivity plays an important role in shaping the genetic structure and the distribution of genetic diversity across the seascape. Connectivity primarily occurs through the dispersal and exchange of planktonic larvae (Green et al., 2015; Leis, 1991), which depends on the oceanographic and geographic features of the seascape, and the behavior of larvae (e.g., Bode et al., 2019; Gaither et al., 2011) and spawning adults (Ma et al., 2018). Meanwhile, the efficacy of larvae to recruit to an appropriate habitat, survive, reproduce, and ultimately, contribute to gene flow, will depend on pre‐ and post‐settlement selection. When selection is strong or persistent, the gradual accumulation of adaptive differences may act to re‐enforce demographic boundaries and create a scenario where migrants are maladapted to the environment that they recruit to Orsini et al. (2013). In some cases, spatio‐temporal variation in these processes can result in chaotic patterns of genetic structure that may be difficult to explain (i.e., chaotic genetic patchiness; Johnson & Black, 1982).

Historical events can also have a profound impact on the demography of populations, and it is important to recognize their legacy on contemporary genetic diversity. For instance, changes in sea level during glacial periods have modified the distribution of shallow coral reef habitat over the last several hundred thousand years, leading to the formation and regression of physical barriers and periods of isolation often followed by secondary contact (e.g., Gaither et al., 2011; Rocha et al., 2007). Changes in sea level can also trigger population bottlenecks or range expansions and founder events (e.g., Avise, 2009), which may decrease effective population size and increase the rate of genetic drift from ancestral populations. Together, these historical and contemporary processes work in concert to culminate in complex patterns of genetic structure and diversity across the seascape. Nonetheless, they must be implicitly recognized in order to fully understand how diversity is generated and the relative efficacy of populations to adapt to changing environmental conditions.

**FIGURE 1:**
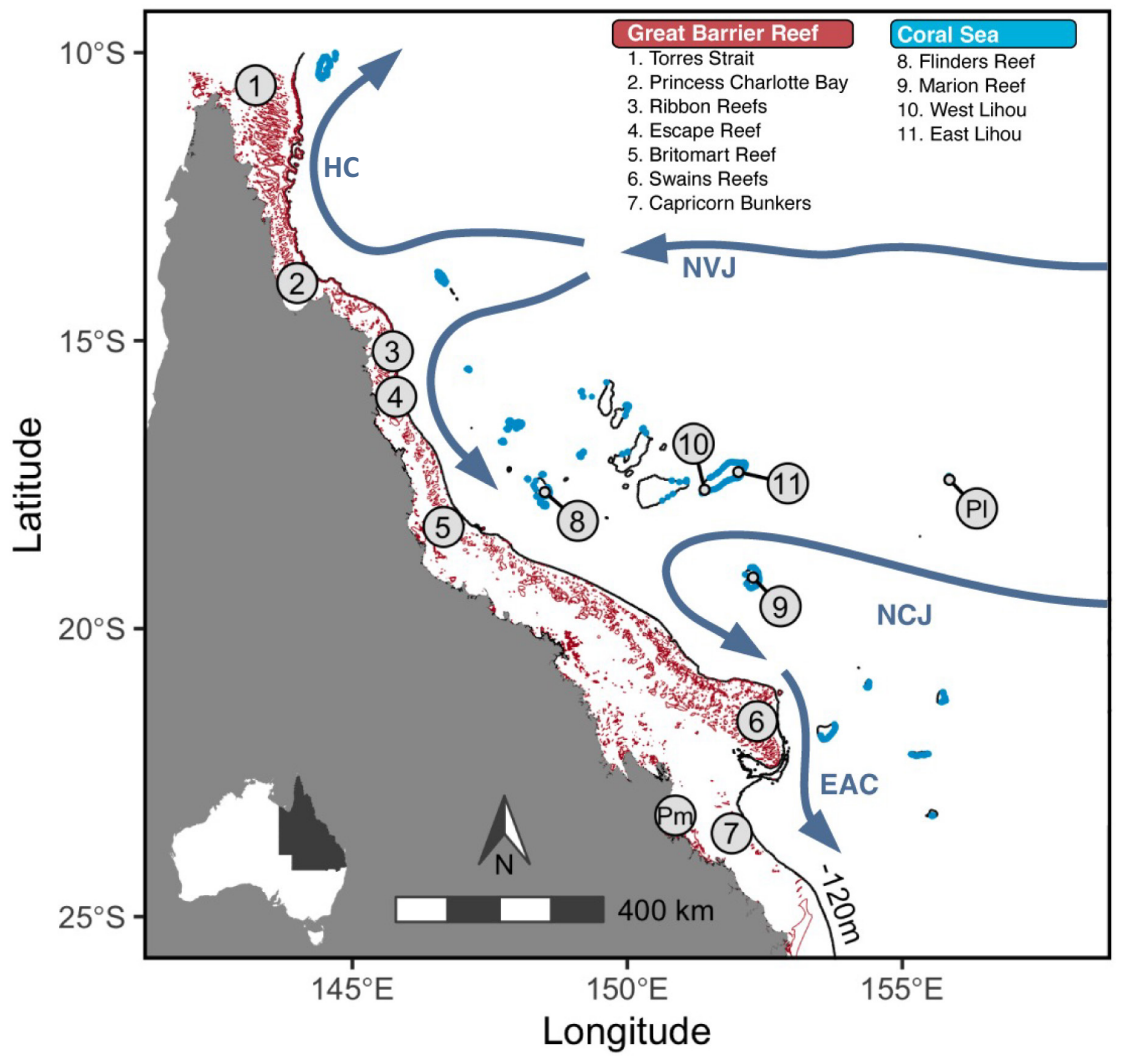
Sampling locations for *Plectropomus leopardus* in the Great Barrier Reef (GBR; red reefs) and the Coral Sea (blue reefs). Sampling locations for reference collections of congeneric species; *Plectropomus laevis* and *P. maculatus* are shown as Pl and Pm, respectively. All reference samples were collected at locations where *P. leopardus* do not occur or from samples previously identified to be “pure” individuals (Harrison, Berumen, et al., 2017). The Southern Equatorial Current is considered the dominant oceanographic feature in the region and moves west through the Coral Sea as the North Vanuatu Jet (NVJ) and the New Caledonia Jet (NCJ), before bifurcating on the central GBR as the north flowing Hiri Current (HC) and south flowing East Australia Current (EAC). The solid black line shows the 120 m depth contour indicating the lowest approximate water level in the GBR and the Coral Sea during the last two glacial periods between 190–130 kya and 10–120 kya. Currents are re‐drawn from Burrage (1993) and Ceccarelli et al. (2013).

Coral reefs within the adjoining Great Barrier Reef (GBR) and the Coral Sea regions in eastern Australia are characterized by unique geological histories and environments, that are likely to have shaped the evolution of respective coral reef communities (Ceccarelli et al., 2013). The GBR currently encompasses a network of 2900 coral reefs that extend approximately 2200 km along the eastern margin of the Australian continental shelf. Sea level fluctuations over the last 400,000 years have seen repeated exposure of shelf‐habitat and presumable relegation of shallow coral reef fauna to a narrow strip of the fringing reef on the outer margin of the shelf (Davies et al., 1989; Veron, 2008). The present configuration of the GBR was established approximately 10 kya following a rapid rise in sea level, which saw the re‐establishment of coral reef species in previously exposed habitat (Davies et al., 1989; Veron, 2008). In contrast, the Australian Coral Sea Marine Park (herein referred to as the Coral Sea), which borders the GBR to the east, and contains 37 coral reef atolls, has provided a comparatively stable habitat over similar evolutionary time scales as the region is characterized by a deep oceanic basin that prevented complete exposure during lower sea level stands (Figure 1). Reefs in the Coral Sea also occupy unique temperature regimes (Payet et al., 2020) and are surrounded by well‐mixed, oligotrophic waters that are far removed from the coastal rainfall, nutrient and sediment runoff that influence the marine environment in the GBR.

There are several oceanographic features within the GBR and Coral Sea that may mediate larval exchange and gene flow between regions. The Southern Equatorial Current is considered the dominant oceanographic feature in the region (Ceccarelli et al., 2013) and flows west through the Coral Sea as the North Vanuatu Jet and the New Caledonia Jet (Figure 1). As they approach the Australian continental shelf, these jets bifurcate and become the Hiri Current flowing to the north and the East Australian Current flowing to the south (Figure 1; Burrage, 1993; Ceccarelli et al., 2013; Kessler & Cravatte, 2013). Along the length of the GBR, high levels of genetic connectivity have been observed among species of reef fish (Evans et al., 2010; van Herwerden et al., 2009) and invertebrates (Harrison, Pratchett, et al., 2017), which has been attributed to the close network of reefs and multidirectional currents that facilitate larval transport. There are, however, a number of species that display a break in genetic structure or diversity between southern and northern GBR latitudes, including sea snakes (Lukoschek et al., 2007), giant clams (Benzie, 1991; Macaranas et al., 1992), echinoderms (Benzie, 1994), corals (van Oppen et al., 2011), and reef fishes (Doherty et al., 1995). These patterns are believed to reflect postglacial re‐establishment of populations in the GBR approximately 10 kya, via refugia located in the Queensland Plateau and the Marion Plateau, in the Coral Sea (Benzie, 1994; van Oppen et al., 2011). Yet, despite these inferences, no study has measured genetic connectivity across the GBR and Coral Sea in a larval dispersing species, with the Coral Sea remaining largely understudied (Ceccarelli et al., 2013).

The common coral grouper (*Plectropomus leopardus*) is widely distributed through coral reefs in the Indo‐West Pacific and is ubiquitous in the GBR (Frisch et al., 2016). Like most species of reef fishes, *P. leopardus* are broadcast spawners, and their larvae are capable of dispersing at least 250 km during their extended (26 days) pelagic larval duration (Williamson et al., 2016). As a predatory species that occupies a high trophic level, they play an important role in shaping the structure of local fish communities (Boaden & Kingsford, 2015; Graham et al., 2003). However, overfishing has led to declines in coral grouper populations throughout most of their range (Sadovy de Mitcheson et al., 2013), and the loss of live coral cover and reef complexity due to an increasing frequency and severity of storms, the crown of thorns starfish outbreaks and coral bleaching events have further compounded these threats (Brown et al., 2021; Emslie et al., 2015; Williamson et al., 2014). In the GBR, coral grouper account for the largest annual catch within the Coral Reef Fin Fish Fishery (725–850 *t*) due to their high value in the international export market (BDO EconSearch, Economic and Social Indicators for the Queensland Coral Reef Fin Fish Fishery, 2020; Queensland Department of Agriculture and Fisheries, Queensland Fisheries Summary, 2018). The GBR stock is currently managed as 12 spatial subpopulations, and groupings are defined based on habitat and consideration of supporting census data (Campbell et al., 2019), though there is a lack of information on the genetic stock structure using modern molecular methods.

In the Coral Sea, *P. leopardus* is found on several isolated atoll reefs, and while these populations are relatively unfished at present (Payet et al., 2020), the extent of connectivity with the GBR and the relative reliance on self‐recruitment is not known. Cross‐shelf patterns of the genetic structure are also rarely considered in fisheries management, despite an increasing number of studies identifying distinct inshore and offshore ecotypes in marine organisms (e.g., Atlantic cod; Barth et al., 2017, European anchovy; Bonhomme et al., 2022, Gulf pipefish; Flanagan et al., 2021, seahorses; Riquet et al., 2019, and dolphins; Louis et al., 2021). Therefore, the aim of this study was to determine the population genomic structure of *P. leopardus* in the Coral Sea and along the length of the GBR using a genotype‐by‐sequencing approach. We also inferred the demographic histories of populations to determine whether historical changes in sea level have influenced the contemporary patterns of genetic structure and genetic diversity. Our findings inform how spatial and temporal processes shape the population genomics of an ecologically important fishery species, with implications for management and conservation.

## 2 METHODS

### 2.1 Sampling

Tissue samples from *P. leopardus* individuals were collected from all reefs where they occur in the central Coral Sea. These were Flinders Reef and two locations at Lihou Reef (Lihou West and Lihou East) on the Queensland Plateau, and Marion Reef on the Marion Plateau (Figure 1). *Plectropomus leopardus* also occur on Saumarez Reef and Wreck Reef in the southern Coral Sea, and Osprey Reef and Ashmore Reef in the northern Coral Sea; however, sufficient sample sizes could not be collected at these locations due to the relative rarity of *P. leopardus* individuals. In the GBR, samples were collected at seven locations from the Torres Strait in the north to the Capricorn Bunkers in the south (Figure 1). A total of 231 skin or fin‐clip samples of *P. leopardus* were collected between 2015 and 2019 using primarily nonlethal biopsy probes (method described by Evans, 2008) but also hook‐and‐line fishing and spear guns.

Because *P. leopardus* are observed to hybridize with congeneric species where they co‐occur (e.g., *Plectropomus maculatus*, Harrison, Berumen, et al., 2017), we also screened for putative hybrids in our collections in order to not confound population genetic analyses. To identify hybrids, we collected 10 reference samples of *P. laevis* from Mellish Reef (Figure 1), where *P. leopardus* are not known to occur, and obtained 10 samples of *P. maculatus* from the southern GBR that were previously identified as purebred individuals by Harrison, Berumen, et al. (2017). All tissue samples were immediately preserved in 95% ethanol and stored at −5°C, and ethanol was replaced with fresh 95% ethanol within 14 days. Samples were collected under the Great Barrier Reef Marine Parks permit No. G11/3354.1 and G17‐39438.1, Queensland General Fisheries permit No. 148534 and 187,992, Parks Australia permit No. AU‐COM2016‐304, AU‐COM2016‐335, and AU‐COM2016‐394, and James Cook University Animal Ethics Permit A1625 and A2273.

### 2.2 Genomic library preparation

Tissue was subsampled and DNA extracted using the NucleoSpin Tissue Kit (Macherey‐Nagel). Eight genomic libraries were prepared following the double digest RADseq protocol (ddRAD, Peterson et al., 2012). Each library contained 80 individuals, and samples were prepared alongside other *Plectropomus* collections not used in this study. First, DNA extractions were quantified using the Qubit HS dsDNA assay kit (Life Technologies) and a Qubit 2.0 fluorometer and standardized to 200 ng gDNA before digesting for 3 h at 30°C using the restriction enzymes SphI and MluCl (New England BioLabs). Digested gDNA was cleaned with Dynabeads™ M‐270 Streptavidin (Life Technologies), quantified using the Qubit HS dsDNA assay kit (Life Technologies) and a Qubit 2.0 fluorometer, and standardized to 60 ng gDNA. Universal P2 adapters were then ligated onto fragment ends, and sets of 16 individuals were barcoded with unique P1 adapters. Individuals were pooled into groups of 16 and bead cleaned before selecting the 450–500 bp fragment size using the Pippin Prep (Sage Science). Unique Illumina indices were added to their respective pools, and gDNA was amplified with 10 PCR cycles using the high‐fidelity Platinum™ Taq DNA Polymerase (Thermo Fischer Scientific). The molarity of the pools was quantified using the High Sensitivity Qubit Kit and the 2100 Bioanalyzer (Agilent Technologies). For each library, we combined pools into a single standardized sample, and 150 bp paired‐end reads were sequenced on a single lane of an Illumina HiSeq 4000. Sequencing was conducted by the King Abdullah University of Science and Technology Bioscience Core Laboratory, Saudi Arabia.

### 2.3 Admixture and species identification

Hybrid fishes can be difficult to identify based on color alone so we ran a series of tests to screen for admixed individuals in our collections. Data were assembled de novo using Stacks version 2.3.5 (Catchen et al., 2013) with the James Cook University High Performance Computer cluster. Paired‐end reads were de‐multiplexed using the “*process_radtags.pl*” pipeline with combinatorial P1 and P2 barcodes, discarding raw reads with an average Phred quality score of less than 30 across a sliding window of 75 bp. To minimize allelic dropout and maximize the number of informative loci available to detect admixed individuals, data were parsed into batches of 30 *P. leopardus* individuals with 10 reference individuals of either *P. laevis* or *P. maculatus*. Each dataset was then filtered using the “*denovo_map.pl*” pipeline with default settings for the parameters *M* (number of mismatches allowed between stacks within individuals; 2), *n* (number of mismatches allowed between stacks between individuals; 3), and *m* (minimum depth of coverage required to create a stack; 3). For each dataset, we used the *population* *s* function to retain loci with a minimum coverage of 10×, loci in every individual (–*r* = 1), and loci in both populations (i.e., species; −*p* = 2).

We then used the program STRUCTURE version 2.3.4 (Falush et al., 2007; Pritchard et al., 2000) to estimate individual admixture proportions. Each STRUCTURE run had a burn‐in of 50,000 MCMC iterations, and 100,000 iterations were retained. No prior information on populations was used, and the number of putative populations, or species (*K*), was set to two. Three replicate runs were conducted for each *K*, and admixture proportions were averaged across replicates. We used a conservative approach and excluded any *P. leopardus* individuals with an admixture proportion greater than 1%.

### 2.4 De novo assembly and filtering

Raw reads for “pure” *P. leopardus* individuals were assembled using similar methods; however, settings for *M* and *n* parameters were optimized following Paris et al. (2017). This approach maximizes the number of informative (i.e., polymorphic) loci while minimizing false positive rates by choosing parameter settings that are most appropriate for levels of polymorphism inherent to the dataset. First, 10 individuals with the least missing data were selected from each population, and stacks were assembled using the *ustacks* function with *M* set at 1 to 10 while holding *n* constant at a default value of 2. The value for *M* was chosen based on the point where the number of new polymorphic loci asymptotes (Figure S1). The optimal value for *n* was parameterised by varying *n* = *M* and *n* = *M* ± 1 in the *ustacks* function, before choosing the *n* value with the maximum number of polymorphic loci (Table S1). The optimal parameter settings for our data were *M* = 5 and *n* = 6. The *m* parameter was set at the default value of 3 as this is shown to be appropriate for datasets with varying levels of polymorphism (Paris et al., 2017).

The full dataset was then assembled de novo, using the parameterised settings for *M* and *n* in the *ustacks* function. To improve computational efficiency, we selected 10 random individuals per population (i.e., sampling location) when implementing the *cstacks* function, as most informative loci are likely to be captured within the first 10 individuals of a population (Rochette & Catchen, 2017). The *populations* function was used to filter for loci that were present in 70% of individuals within every population, and we kept one SNP per read using the *write_single_snp* command.

The resulting dataset was then imported into the R package *Radiator* version 1.2.1 (Gosselin, 2020) for further quality filtering depending on the requirement of the analyses. For assessing population genomic structure we first removed monomorphic loci and set the minimum minor allele count to four. We retained loci with mean coverage between 10× and 100× and excluded markers that had more than 10% missing data. We accounted for potential re‐sampling of released fish by using the *detect_duplicate_genomes* function to identify any duplicate samples with low pairwise genetic distance (<0.1). To test for potential contamination, we then screened for individuals with unusually low (<0.08) or high (>0.12) heterozygosity relative to other individuals in the sample, and for individuals that had more than 30% missing data. Lastly, we excluded loci with heterozygosity >0.5 and markers that were in Hardy–Weinberg disequilibrium in two or more populations using a mid‐*p* value threshold of 0.05.

Outlier loci were identified using the R package *PCAdapt* (Luu et al., 2017) and BayeScan V2.1 (Foll & Gaggiotti, 2008). *PCAdapt* uses principal component analyses (PCA) to compute test statistics and *p* values for each locus based on correlations between SNPs and principal components. We set *K* at 2 as this was identified as the most likely number of clusters in our data (Figure S2) and used a false discovery rate of 10%. For BayeScan, we used default settings, which included conducting 20 pilot runs of 5000 iterations each with a burn‐in length of 50,000, a thinning interval of 10 and a prior odds ratio of 10. Outlier markers identified using either method were excluded from the neutral dataset.

Genomic data for demographic inference were constructed de novo and filtered using the same parameter thresholds including the removal of outlier loci. However, we did not exclude rare alleles (minor allele count) or loci in Hardy–Weinberg disequilibrium, as these are likely to be influenced by demographic processes and are informative for inferring the demographic histories of populations.

### 2.5 Population  structure and diversity

Observed and expected heterozygosity and allelic richness were calculated in the R package *strataG* (Archer et al., 2017). Inbreeding coefficients (*F*_IS_) were estimated using the *divBasic* function in the R package *diveRsity* version 1.9.90 (Keenan et al., 2013), with 95% confidence intervals tested at 1000 bootstrap replicates. To determine if there was significant genetic structures across all sites, we conducted an AMOVA in *strataG* (Archer et al., 2017) and estimated pairwise *F*_ST_ between sites (Weir & Cockerham, 1984) to explore genetic subdivision. Significance of pairwise *F*_ST_ comparisons was estimated via 1000 bootstrap replicates, and the *p* value threshold was corrected for multiple comparisons as per Benjamini and Yekutieli (2001). We used a Discriminant Analysis of Principal Components (DAPC) in the R package *Adegenet* version 2.1.3 (Jombart, 2008) to visualize genetic clustering, using the *optima‐a‐score* command to retain 26 principal components and 10 discriminant functions (*N* populations minus 1).

To explore differences in ancestral genetic diversity, we used sparse non‐negative matrix factorization (SNMF) in the R package *LEA* (Frichot & François, 2015). SNMF estimates admixture coefficients by producing a least‐squares estimate of ancestral (i.e., source) populations at *K* ancestral populations, producing comparable results to similar methods (e.g., STRUCTURE, Pritchard et al., 2000) with significantly lower computational time (Frichot et al., 2014). We ran 20 replicates with the putative number of ancestral populations (*K*) from 1 to 11. The most parsimonious number of populations (*K*) was determined by selecting the run with the lowest cross‐entropy criterion (Figure S3). We conducted similar analyses with outlier markers including pairwise *F*_ST_ estimates, SNMF and DAPC. For DAPC, we used the *optima‐a‐score* command to retain 21 principal components and 10 discriminant functions (*N* populations minus 1).

### 2.6 Demographic inference

Glacial periods have influenced sea level through time and have had a significant effect on the demography of coral reef fishes through the Indo‐West Pacific (Avise, 2009; Hewitt, 2004; Hickerson et al., 2010). To determine whether these events have influenced the evolutionary history of *P. leopardus* in the Coral Sea and the GBR, we inferred the demographic histories of populations using a modified diffusion approximation of the Wright‐Fischer population model implemented in the program GADMA (Noskova et al., 2020). GADMA infers the joint demographic history of up to three populations from genetic data and has benefits over other programs because it does not require the user to specify the large number of possible demographic histories that may characterize a group of populations. GADMA allows the user to choose between two common diffusion approximation methods: ∂a∂I (Gutenkunst et al., 2009) and *moments* (Jouganous et al., 2017) and implements a fitness function to explore the parameter space based on the principles of “natural selection.” Briefly, an initial set of models is randomly generated and is mutated by randomly changing values of model parameters or crossed over by randomly selecting parameters from two different models to generate a new model.

We observed two geographically distinct genetic clusters in our data; the Coral Sea and the GBR, and inferred the demographic histories of each of these regions using two replicate site comparisons. Sites with the largest sample sizes were selected in order to minimize biases in the calculation of the joint site frequency spectrums (JSFSs). We chose to compare Britomart (GBR) vs. Flinders (Coral Sea) due to their geographic proximity and Princess Charlotte Bay (GBR) vs. East Lihou (Coral Sea) to determine whether results were consistent at a broader spatial scale.

Joint site frequency spectrums for each pair of sites were generated from variant call format (VCF) files using the easy SFS python script (https://github.com/isaacovercast/easySFS). We used one SNP per locus, and thus loci were assumed to be unlinked and 1,060,096 bp as the effective sequence length. An empirical mutation rate is not known for our study species, so we chose to use the mutation rate of 1.0E^−08^, which is most commonly reported for fishes (Jacobs et al., 2018; Le Moan et al., 2016; Rougeux et al., 2017; Souissi et al., 2018; Tine et al., 2014), resulting in a model theta of 0.424 mutations per site per generation. We used a generation time (*t2*) of 8.25 years based on the formula by Pianka (1978): *t2* = (*a* + *w*)/2, where *a* = mean age (years) at 50% maturity and w = longevity (years). We set *a* at 2.5 years and *w* at 14 years based on the known life history of *P. leopardus* in the GBR (Adams et al., 2000; Ferreira & Russ, 1994; Russ et al., 1998) and the Coral Sea (Payet et al., 2020).

*Moments* was selected as the software to simulate allele frequency spectra from demographic models because it has shown to produce robust results and is computationally more efficient than alternatives (Noskova et al., 2020). Initial model structure was set to one time interval before the population split and one after (1,1). Final model structure was also set to 1,1. No bounds for divergence times were included. Parameters for the Genetic Algorithm (GA) were left as default as recommended by the developers of the program; these parameters have been tested and validated for different datasets and demographic scenarios (for details see Noskova et al., 2020). Powell's conjugate direction method was selected for local model optimization after each genetic algorithm. Models were drawn every 100 iterations, and 10 independent replicates were run for each dataset. The Akaike Information Criterion (AIC) was used for model comparison and selection as implemented in GADMA.

In addition to these models, we ran a series of more complex demographic scenarios to include one time interval before the split and two time intervals after the split (1,2). This allowed us to test for consistency across models, while acknowledging that these results should be interpreted with caution because inferring complex scenarios using a relatively small number of loci (i.e., 1000's) of unknown evolutionary history may result in over‐fitting of the data. Together, the complex and simple models captured many possible demographic scenarios, including the four most common that are often inferred from genomic data: strict isolation (SI), ancient migration (AM), isolation with migration (IM), and secondary contact (SC). The list of models and parameter space of the 20 best models for each dataset are shown in Tables S2 and S3.

## 3 RESULTS

### 3.1 Quality control

A total of 31 individuals were excluded due to having evidence of mixed ancestry >1%, all admixed individuals were sampled on reefs where they co‐occur with congeneric species (Table S3), highlighting the importance of considering hybridisation when resolving population genetic structure.

The remaining 200 *P. leopardus* samples passed further quality filtering steps, and no individuals were excluded while screening for duplicate samples, individual heterozygosity, and individual missing data. We retained 4548 neutral markers for analyses of population genomic structure, 230 outlier markers (of which 42 were detected by both methods), and 10,479 markers for inference of demographic histories. Parameter settings and the corresponding number of filtered loci are shown in Table S4.

### 3.2 Population structure and diversity

**FIGURE 2:**
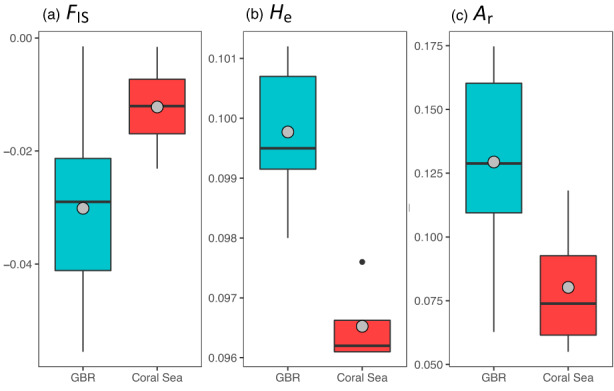
Regional summaries of (a) the inbreeding coefficient (*F*_IS_), (b) genetic diversity (*H*_e_), and (c) allelic richness (*A*_r_). Horizontal black lines indicate the median, and gray circles indicate the mean. Upper and lower boundaries of the box indicate 75% and 25% quartiles, respectively, while outliers are shown as black circles.

Regional differences in diversity indices were observed between the GBR and the Coral Sea. All sites had negative estimates of the inbreeding coefficient (*F*_IS_) suggesting recent expansions or mixing; however, *F*_IS_ estimates were closer to zero in the Coral Sea (Table 1, Figure 2). Consistent with this pattern, sites in the Coral Sea had lower genetic diversity according to estimates of heterozygosity (*H*_e_) and allelic richness (*A*_r_; Table 1, Figure 2).

**TABLE 1:** Sample sizes and genetic diversity indices for *Plectropomus leopardus* sites sampled in the Great Barrier Reef and the Coral Sea

Region	Site	*N*	*N*_SNPs_	*H*_*o*_	*H*_*e*_	*A*_r_ (±SER)	*F*_IS_ (95% CI)
Great Barrier Reef	Torres Strait	9	6576	0.0980	0.0901	0.1747 ± 0.0009	**−0.0521** (−0.1405 to −0.0042)
	Princess Charlotte Bay	29	7909	0.0994	0.0933	0.0628 ± 0.0002	−0.0015 (−0.0177 to 0.0122)
	Ribbon Reefs	13	7051	0.1011	0.0939	0.1288 ± 0.0006	**−0.0283** (−0.0769 to −0.0019)
	Escape Reef	9	6658	0.0995	0.0935	0.1743 ± 0.0009	**−0.0555** (−0.1365 to −0.0061)
	Britomart Reef	18	7464	0.1003	0.0922	0.0970 ± 0.0004	−0.0143 (−0.0478 to 0.0086)
	Swains Reefs	14	7086	0.0989	0.0898	0.1219 ± 0.0006	−0.0302 (−0.083 to 0.0036)
	Capricorn Bunkers	11	6853	0.1012	0.0929	0.1462 ± 0.0008	−0.0290 (−0.0895 to 0.0073)
Coral Sea	Flinders Reef	20	7423	0.0976	0.0921	0.0841 ± 0.0004	−0.0016 (−0.0238 to 0.0144)
	Marion Reef	14	6982	0.0961	0.0876	0.1182 ± 0.0006	−0.0231 (−0.0654 to 0.0054)
	Lihou East	34	7850	0.0963	0.0890	0.0550 ± 0.0002	**−0.0149** (−0.0284 to −0.0042)
	Lihou West	29	7670	0.0961	0.0875	0.0637 ± 0.0003	−0.0092 (−0.0286 to 0.0066)

*Note*: *N*, number of individuals sampled; *N*_SNPs_, number of single nucleotide polymorphisms; *H*_o_, observed heterozygosity; *H*_e_, expected heterozygosity; *A*_r_, allelic richness ± standard error; *F*_IS_, inbreeding coefficient with 95% confidence intervals estimated via 1000 bootstrap replicates; bold indicates significant negative *F*_IS_ estimates.

**FIGURE 3:**
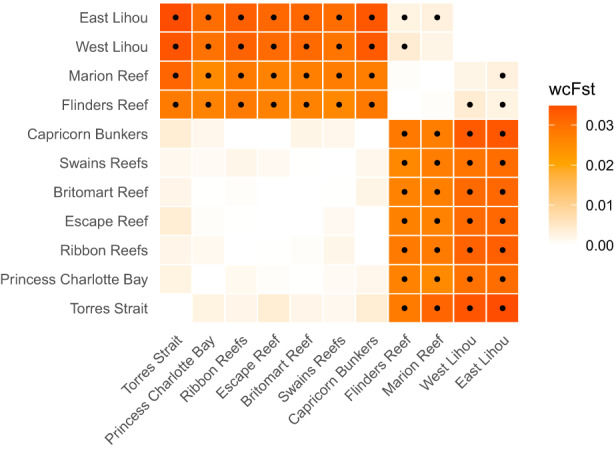
Pairwise *F*_ST_ (Weir & Cockerham, 1984) heat map for *Plectropomus leopardus* collected in the Great Barrier Reef and the Coral Sea based on 4548 neutral SNP markers. Pairwise comparisons that were significantly different from zero at a corrected alpha threshold of 0.011 (Benjamini & Yekutieli, 2001) are indicated by a black circle. SNP, single nucleotide polymorphism.

**FIGURE 4:**
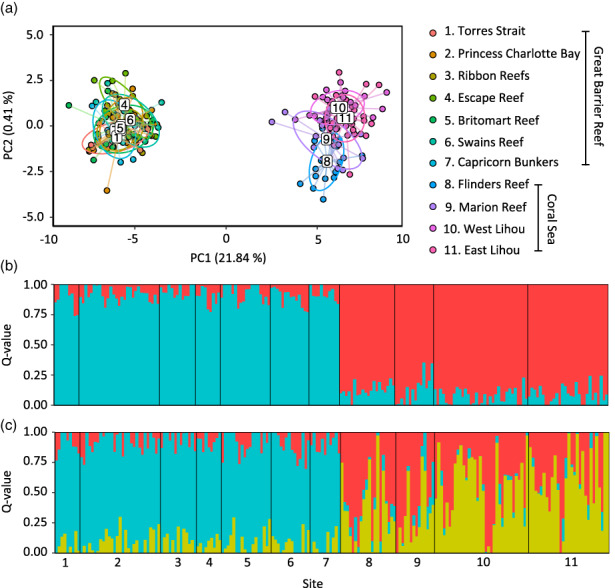
(a) Scatterplot of discriminant analyses of principal components (DAPC, *Adegenet* v2.1.3, Jombart, 2008) for populations of *Plectropomus leopardus* sampled in the Great Barrier Reef (GBR) and Coral Sea based on 4548 neutral SNP markers, explaining 22% of total variance in the genetic data. Bar plots of admixture coefficients for *P. leopardus* at (b) *K* = 2 and (c) *K* = 3 estimated using sparse non‐negative matrix factorization (SNMF) in the R package *LEA* (Frichot & François, 2015). Each vertical bar represents an individual, and the color indicates relative admixture coefficients at *K* ancestral populations. Populations are separated by black vertical lines and labeled according to the legend. GBR populations are ordered north to south from left to right, and Coral Sea populations are ordered by proximity to the GBR, from left (closest) to right (farthest). SNP, single nucleotide polymorphism.

Analyses of molecular variance (AMOVA) identified significant genetic structure among *P. leopardus* in the Coral Sea and the GBR (Global *F*_ST_ = 0.017, *p* < 0.001). Pairwise *F*_ST_ comparisons revealed a significant genetic break between all sites in the Coral Sea versus all sites in the GBR (Figure 3). Similarly, DAPC identified strong regional genetic clustering, which accounted for 21.8% of variance along the first principal component (Figure 4a) and 22% of total variance. SNMF analyses indicated strong regional divergence (Figure 4b), and the most parsimonious number of ancestral clusters (*K*) in our data was two (Figure S3).

The greatest genetic distance between Coral Sea and GBR *P. leopardus* sites was between Torres Strait (the northern‐most site sampled in the GBR) and Lihou East (the eastern‐most reef sampled in the Coral Sea; pairwise *F*_ST_ = 0.035, *p* < 0.001). The two most genetically similar sampling locations between regions were Marion Reef in the Coral Sea and Princess Charlotte Bay in the GBR (pairwise *F*_ST_ = 0.025, *p* < 0.001).

In the GBR, we found no evidence of genetic structure (Figures 3 and 4), indicating high levels of genetic connectivity in the region. According to DAPC and SNMF analyses, there was evidence of genetic structure between all sites in the Coral Sea, with the exception of the east and west sites sampled at Lihou Reef (Figures 3 and 4a, c). The maximum pairwise *F*_ST_ in the Coral Sea was between Flinders Reef and Lihou West (*F*_ST_ = 0.004, *p* < 0.001) while the smallest pairwise *F*_ST_ in the Coral Sea was between Lihou West and Lihou East (*F*_ST_ < 0.001, *p* = 0.663).

Outlier markers revealed broadly similar results to neutral loci, with a strong regional divergence between GBR and Coral Sea regions (Figures S4 and S5) and a maximum pairwise *F*_ST_ estimate of 0.23 (*p* < 0.001). Within the GBR, there was evidence of structure between some sites according to pairwise *F*_ST_ comparisons (Figure S4), although this was not corroborated by results from DAPC or SNMF analyses (Figure S4).

### 3.3 Demographic inference

**FIGURE 5:**
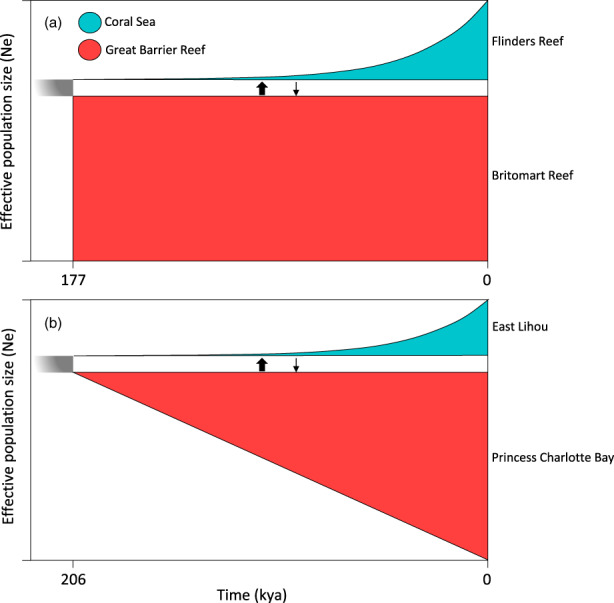
Best‐ranked demographic models describing divergence and changes in population size between (a) Flinders Reef (Coral Sea) and Britomart Reef (Great Barrier Reef) and (b) East Lihou (Coral Sea) and Princess Charlotte Bay (Great Barrier Reef). Demographic models were constructed using the diffusion approximation method (*moments*; Jouganous et al., 2017) implemented in the program GADMA (Noskova et al., 2020). Joint site frequency spectra for empirical and inferred data are shown in Figure S1.

We identified the regional genetic structure between Coral Sea and GBR *P. leopardus* and inferred the demographic histories of two population pairs (i.e., replicates); Flinders (Coral Sea) vs. Britomart (GBR), and East Lihou (Coral Sea) vs. Princess Charlotte Bay (GBR). For both simple model replicates, the best‐ranked model identified population divergence via isolation with asymmetric migration (Tables S2 and S3; Figures 5 and S6). The estimated timing of divergence from the GBR for East Lihou was 206 kya and for Flinders Reef was 177 kya (Figure 5). Estimated migration rates were an order of magnitude higher from the GBR to the Coral Sea for both comparisons. Migration rates from Britomart Reef to Flinders Reef were 3.7 × 10^−4^, whereas migration from Flinders Reef to Britomart Reef was 4.2 × 10^−5^. For the second population pair, migration from Princess Charlotte Bay to East Lihou was 3.55 × 10^−4^, whereas migration in the inverse direction was 3.35 × 10^−5^.

Because the population expansion at Britomart Reef on the GBR was sudden, we do not interpret ancestral population size estimates from this model (Noskova et al., 2020). For the Princess Charlotte Bay versus Lihou East model, the effective size of the ancestral population was two orders of magnitude smaller in the Coral Sea than in the GBR (41 versus 4115, respectively). Population expansions after divergence were observed in both regions (Figure 5). In the Coral Sea, expansions were exponential with contemporary effective population sizes of 18,013 and 25,947 at East Lihou and Flinders Reef, respectively. In the GBR, contemporary effective population sizes were consistently larger than the Coral Sea at 51,600 and 83,137 for Britomart Reef and Princess Charlotte Bay, respectively. Population expansions at Princess Charlotte Bay were linear, whereas expansions at Britomart Reef were sudden (Figure 5).

Complex model comparisons resulted in a slightly better fit to SFSs than simple models (Tables S2 and S3). The best fit model for each replicate comparison indicated a period of constant population size after population split, followed by population expansion (5 kya for Britomart Reef vs. Flinders Reef and 12 kya for East Lihou vs. Princess Charlotte Bay; Figures S7 and S8). The estimated timing of divergence was similar to the simple models, albeit slightly later (160 and 163 kya). Migration rates were also asymmetric and similar in magnitude to those inferred in the simple models, with higher relative migration from the GBR to the Coral Sea during the period of constant population size and during the period of population expansion (Table S3, Figure S7). The complex models also indicated that the Coral Sea was founded by a small number of individuals, has experienced a recent population expansion, and has a contemporary effective population size that is smaller than the GBR (Table S3, Figure S7).

## 4 DISCUSSION

We assess the population genomic structure of a commercially and ecologically important species of coral reef fish (*P. leopardus*) across 14 degrees of latitude and 9 degrees of longitude in the west Pacific region of Australia. We find limited genetic connectivity between *P. leopardus* in the GBR and the Coral Sea despite the proximity of these two regions (~100 km). Inferred demographic histories for two replicate comparisons suggest that divergence occurred under a model of isolation with migration and was initiated at approximately 206 and 177 kya, with one model comparison identifying an ancestral population in the Coral Sea that was two orders of magnitude smaller than the GBR, in terms of effective population size (*N*_e_). Expansions were evident in both regions despite concurrent changes in sea level, but genetic diversity and contemporary effective population sizes (*N*_e_) were smaller in the isolated atolls of the Coral Sea. We found high levels of gene flow along the majority of the GBR and significant genetic structure within the Coral Sea. Our results indicate a unique evolutionary history of *P. leopardus* in each region.

### 4.1 Evolution of independent lineages

Divergence of GBR and Coral Sea populations appears to predate the most recent glacial period that would have seen the exposure of the GBR shelf from 120 to 10 kya. The imprint of the associated glacial maximum (~20kya) on the demography of coral reef fishes is well documented in the literature (DiBattista et al., 2016; Knowlton & Weigt, 1998; Ludt & Rocha, 2015; Rocha et al., 2007), though we did not find that it was responsible for the divergence of *P. leopardus* lineages in this study. Instead, we show that divergence is likely to have occurred during the early stages of the preceding glacial period from 190 to 130 kya. This finding suggests that *P. leopardus* in the Coral Sea have been isolated from the GBR for a significant period of time and that these lineages have maintained some level of independence despite concurrent changes in sea level.

Estimated migration rates were 10 times higher from the GBR to the Coral Sea, and ancestral population sizes in the latter were two orders of magnitude smaller, according to one of the model comparisons. This suggests that the Coral Sea population was likely established by a small number of individuals from the ancestral population (i.e., founder effect). It is possible that changing environmental conditions in the Coral Sea (e.g., availability and/or quality of coral habitat) facilitated expansion into the region and that these conditions remained favorable enough to support populations during the ~190 ky period that followed. Indeed, *P. leopardus* show strong habitat specificity and are dependent on live coral (or the structural complexity provided by live coral) at the recruit, juvenile and adult life history stages (Emslie et al., 2014; Kerry & Bellwood, 2012; Light & Jones, 1997). In the Coral Sea, *P. leopardus* is found on only seven of 37 reefs; all of which contain a back‐reef or lagoon habitat akin to the mid‐shelf reefs where they are most abundant in the GBR (Frisch et al., 2016). Similarly, in Western Australia, *P. leopardus* occur on coral atolls with back‐reef habitat (i.e., South Seringapatam Reef at −14° latitude and the Houtman Abrolhos Islands at −28° latitude) and are rare or absent in surrounding coral reef habitat (van Herwerden et al., 2009). Hence, changes in habitat are likely to have influenced the distribution of *P. leopardus* through time and may have facilitated the expansion and persistence of populations in the Coral Sea.

Alternatively, the abrupt decline in sea level approximately 190 kya may have reduced available habitat in the Coral Sea, resulting in a population bottleneck that triggered faster relative genetic drift from the ancestral population. This may have been re‐enforced by geographic isolation if the ancestral population was restricted to the southern GBR, as suggested for some coral reef species during periods of low sea level (Benzie, 1994; Doherty et al., 1995; Lukoschek et al., 2007; Macaranas et al., 1992; van Oppen et al., 2011). While it is difficult to determine underlying events that initiated divergence between the Coral Sea and GBR *P. leopardus*, we provide evidence of a potentially complex evolutionary history influenced by factors in addition to changes in sea level.

Expansions were detected for all populations where demographic histories were inferred; however, estimates of contemporary effective population size were significantly smaller in the Coral Sea. Coral Sea sites also had lower genetic diversity and estimates of the inbreeding co‐efficient that were closer to zero. This may be a consequence of a small ancestral founder population in the Coral Sea, the geographic isolation of these reefs, local population substructuring and the smaller habitat area here, compared with the GBR. Core‐edge effects can also contribute to these patterns where selection is stronger in fringing populations. Theoretical and empirical examples show that the effective size of a population should be positively correlated with the time since ancestral divergence (Braasch et al., 2019; Excoffier et al., 2009), and yet, we observed smaller estimates of *N*_e_ at East Lihou despite this population diverging some 29 kya earlier than the Flinders Reef population. The geographic isolation of Lihou Reef appears to impose some limit on larval connectivity, and this may explain the limited increase in *N*_e_ relative to Flinders Reef, which is closer to the GBR. Despite evidence of genetic isolation, expansions were exponential at both Coral Sea reefs and may be a consequence of similar events, potentially initiated by global processes such as changes in sea level (e.g., Delrieu‐Trottin et al., 2020).

Contemporary estimates of *N*_e_ were larger at both GBR sites, and expansions were sudden at Britomart Reef. Habitats in the southern GBR and southern Coral Sea are hypothesized to have provided refuge for coral reef fauna during low sea level stands (Benzie, 1994; Doherty et al., 1995; Lukoschek et al., 2007; Macaranas et al., 1992; van Oppen et al., 2011), and the relative proximity of Britomart Reef to this region may explain the sudden expansion compared with Princess Charlotte Bay in the northern GBR.

Ultimately, we apply a degree of caution when interpreting the precise timing of coalescence using genes with unknown mutation rates and where generation times of focal species are estimated, even though these were sourced from empirical life‐history data in our study (Adams, 2002; Ferreira & Russ, 1994; Payet et al., 2020; Russ et al., 1998). Comparisons involving the complex models provided support for a similar mode of divergence (isolation with asymmetric migration), with recent expansions in both regions (5 and 12 kya) that align with the approximate age of the GBR in its current configuration (Davies et al., 1989; Veron, 2008). While these models provide a better fit for our data, we interpret them with caution. Nonetheless, both simple and complex models reveal general consistencies in the timing of divergence, the direction of migration, and effective population size estimates and provide further evidence of a complex evolutionary history of *P. leopardus* during Pleistocene glaciations (e.g., Ma et al., 2018; van Herwerden et al., 2009). It is not known if our results reflect a broader phylogeographic history across other marine taxa in the Coral Sea as no other study has used similar methods to assess genetic connectivity across the region. Comparisons using a variety of molecular techniques suggest that results are likely to be species‐specific (Benzie, 1991; Benzie & Williams, 1992; Planes et al., 2001).

### 4.2 Contemporary patterns of connectivity

Discrete populations of *P. leopardus* in the Coral Sea and the GBR were identified in all analyses of genetic structure indicating that contemporary connectivity between regions is minimal. This was surprising given that (1) the New Caledonia Jet and the north Vanuatu Jet flow east to west through the Coral Sea and directly intersect the GBR (Figure 1, Burrage, 1993; Ceccarelli et al., 2013; Kessler & Cravatte, 2013), (2) regional populations are as close as 100 km (e.g., Flinders Reef and Britomart Reef), and (3) *P. leopardus* are capable of dispersing over 250 km from their natal reefs (Williamson et al., 2016).

Since the GBR provides a larger habitat area and presumably supports a greater population of *P. leopardus*, it is possible that the genetic signal of any migrants arriving from the Coral Sea is quickly diluted into the local gene pool (i.e., genetic swamping). *Plectropomus leopardus* also form relatively small aggregations (100’s of individuals) at multiple sites (Frisch et al., 2016; Samoilys, 1997) and typically spawn during medium to strong current periods (Zeller, 1998). Ma et al. (2018) provided evidence that these traits may limit the dispersal of *P. leopardus* larvae and explain higher levels of genetic structure in this species relative to other epinepheline (e.g., *E. polyphekadion* and *P. areolatus*) that aggregate in large numbers (1000’s of individuals) and spawn in reef passages adjacent to the open ocean, during high current flow periods. There is also evidence that reef fish populations in habitat‐limited areas (i.e., isolated reefs in the Coral Sea) spawn over narrower time period to maximize local retention of larvae (Boddington et al., 2021; Choat, 2012). In the case of the Coral Sea, this may limit connectivity with the GBR despite the prevailing ocean currents.

Alternatively, larval exchange between regions may occur via planktonic dispersal, with postsettlement processes preventing any realized genetic connectivity. For instance, the gradual accumulation of genetic incompatibility through genetic drift (over some 190 ky) may serve to limit introgression between populations through the evolution of pre‐ or postzygotic reproductive barriers. Though this seems unlikely given that *P. leopardus* are capable of producing fertile hybrids with congenerics (Harrison, Berumen, et al., 2017). Another possible scenario is isolation by adaptation (Orsini et al., 2013), where migrants exchanged between regions have lower rates of survival or reduced reproductive success because they are poorly adapted to the conditions that they recruit to. Indeed, temperature regimes vary significantly between the GBR and the Coral Sea, and there is an overwhelming amount of evidence showing the impact of temperature on the metabolism and behavior of *P. leopardus* (Brown et al., 2021; Johansen et al., 2014, 2015; Pratchett et al., 2017; Scott et al., 2019). While analyses of outlier SNPs showed similar results to the neutral dataset, gene‐environment association tests (e.g., Capblancq & Forester, 2021) may provide a more suitable alternative to determine whether there is a local adaptation in *P. leopardus*.

Within regions, measures of genetic connectivity were generally consistent with the geographic configuration of reefs and the relative continuity of habitat. Similar to previous studies that analyse microsatellite markers (Ma et al., 2018; Taboun et al., 2021) we observed high levels of gene flow in *P. leopardus* across the majority of the 2200 km length of the GBR due to the proximity of individual reefs and the ubiquitous distribution this species. Similarly, sites at East Lihou and West Lihou were genetically homogenous due to the continuous habitat that connects the ~100 km long coral atoll. These findings are consistent with (Pinsky et al., 2012) and highlight that habitat continuity can generate connectivity across large spatial scales (i.e., 100’s to 1000’s km). In the Coral Sea, Lihou Reef was genetically distinct from Flinders and Marion Reefs, which appeared to experience some degree of genetic connectivity with each other. The velocity of the East Australian Current is relatively strong between Flinders and Marion Reefs, and this may facilitate larval connectivity even though the distance (400 km) is well in excess of the maximum documented dispersal of *P. leopardus* larvae (~250 km, Williamson et al., 2016).

### 4.3 Conservation and management implications

Our results add to an increasing body of literature that identifies cross‐shelf patterns of population genetic structure in fishery species with implications for management (e.g., Atlantic cod, Barth et al., 2017; European anchovy, Bonhomme et al., 2022). Coral trout are an iconic fishery species and account for the largest annual commercial catch by volume (725–850 t) and value ($27–$31 m) in the GBR the Coral Reef Fin Fish Fishery (Frisch et al., 2016; Queensland Department of Agriculture and Fisheries, Queensland Fisheries Summary Report, 2018). Our results show that *P. leopardus* in the GBR experience relatively high levels of genetic connectivity but exist as an independent stock to the Coral Sea. Considering that the GBR fishery is split into 12 stock units that align with habitat types (Campbell et al., 2019) and that do not cross any known genetic breaks (i.e., spatial mismatch), the current spatial structure used in stock assessment models appears to be appropriate.

High levels of observed genetic connectivity in the GBR are also likely to benefit the relative resilience of this population because they allow the replenishment of individual reefs from upstream locations. The large effective population size and higher relative genetic diversity may also assist with adaptation to changing environmental conditions. These results, however, should not be interpreted as a panacea, as *P. leopardus* populations have declined throughout the majority of their range (Sadovy de Mitcheson et al., 2013) and are subject to the cumulative impacts of habitat loss, fishing pressure, and climate‐driven thermal stress in the GBR (Brown et al., 2020; Brown et al., 2021; Frisch et al., 2016; Pratchett et al., 2017).

We show that while *P. leopardus* in the Coral Sea escape many coastal impacts due to its distance from mainland Australia such as habitat loss due to coastal runoff (Ceccarelli et al., 2013) and fishing pressure (Payet et al., 2020), populations here represent a unique challenge to managers. The geographic isolation of the Coral Sea and the low level of genetic connectivity between reefs relative to the GBR indicate that populations are primarily reliant on self‐recruitment for replenishment and may be slower to recover should there be local perturbation. *Plectropomus leopardus* are also particularly dependent on live coral cover for habitat (Emslie et al., 2014; Kerry & Bellwood, 2012; Light & Jones, 1997; Williamson et al., 2014) and the spatial and temporal extent of recent coral bleaching events in the Coral Sea (Harrison et al., 2019) and globally (Hughes et al., 2017), generate concern for these isolated populations that occupy a relatively small habitable area. Further, lower genetic diversity and smaller effective population sizes in the Coral Sea may indicate that these populations have less diversity for selection to act upon and a lower relative capacity to adapt to rapidly changing environments (Excoffier et al., 2009).

## 5 CONCLUSIONS

Climate‐driven changes in sea level, their impact on the demography of GBR fauna, and links with the Coral Sea region are often discussed in the literature (Benzie, 1994; Doherty et al., 1995; Lukoschek et al., 2007; Macaranas et al., 1992; van Oppen et al., 2011) but are rarely empirically tested. This study is the first to compare the genetic connectivity and the demographic history of a larval dispersing fish between and within these globally significant regions. We identify spatially discrete genetic lineages with unique evolutionary histories at a scale that is rarely observed in coral reef fishes (i.e., <100 km). While it is difficult to determine the underlying events that triggered the divergence of the Coral Sea and GBR lineages, further research may shed light on the processes that maintain contemporary isolation (e.g., genetic swamping, spawning behavior, adaptation, and genetic drift). Our results demonstrate the importance of considering demographic histories when interpreting population genetic structure and show that this information may provide insight into the relative resilience of isolated populations, particularly those of commercial and ecological significance, such as coral grouper. We add to a growing body of evidence that shows the evolutionary history of widely dispersed marine species can vary at small spatial scales.

## CONFLICT OF INTEREST

The authors declare no conflict of interest.

## Supporting information


Appendix S1

## Data Availability

Individual genotype datasets (Payet, 2022) are available on DataDryad: https://doi.org/10.5061/dryad.np5hqbzwv.
