# Supplementary material for: Demographic histories shape population genomics of the common coral grouper (Plectropomus leopardus)
Source: Evol Appl. 2022 Aug 5;15(8):1221–35. doi: 10.1111/eva.13450 (PMC9423088; doi:10.1111/eva.13450)
Supplement: Supplementary file 1 — Appendix S1 [file EVA-15-1221-s001.docx]

***Supplemental tables***

**Table S1**. Total number of polymorphic loci for each iteration of N.

| N value | Total polymorphic loci |
| --- | --- |
| N = M -1 | 64,406 |
| N = M | 64,529 |
| N = M +1 | 64,724 |

**Table S2.** Summary of the top 20 ranked “simple” model comparisons with model structure set to one time interval before the population split and one time interval after the split (1,1). Models are ordered by rank (1 to 20) according to AIC scores. Flinders Reef vs. Britomart Reef and Lihou East vs. Princess Charlotte Bay are shown in the top and bottom of the table, respectively.

| Flinders Reef (pop 1) vs. Britomart Reef (pop2) | | | | | | | | | | | |
| --- | --- | --- | --- | --- | --- | --- | --- | --- | --- | --- | --- |
| Rank | Log Likelihood | AIC | N ancestral | t1 | nu11 | nu12 | m1_12 | m1_21 | expansion type pop1 | expansion type pop2 | theta |
| 1 | -463.3 | 940.5 | 3118 | 176785 | 25947 | 51600 | 3.68E-04 | 4.21E-05 | Exp | Sud | 132.24 |
| 2 | -463.3 | 940.6 | 2886 | 183160 | 24881 | 51398 | 3.71E-04 | 4.46E-05 | Exp | Sud | 122.41 |
| 3 | -463.4 | 940.7 | 2571 | 189245 | 25136 | 51124 | 3.66E-04 | 4.69E-05 | Exp | Sud | 109.03 |
| 4 | -463.4 | 940.9 | 2492 | 190618 | 26426 | 50729 | 3.53E-04 | 4.71E-05 | Exp | Sud | 105.68 |
| 5 | -463.5 | 940.9 | 2352 | 193687 | 25768 | 50732 | 3.57E-04 | 4.80E-05 | Exp | Sud | 99.74 |
| 6 | -463.5 | 940.9 | 2343 | 193342 | 25642 | 50887 | 3.59E-04 | 4.80E-05 | Exp | Sud | 99.38 |
| 7 | -463.5 | 940.9 | 2342 | 193259 | 25474 | 50683 | 3.61E-04 | 4.71E-05 | Exp | Sud | 99.33 |
| 8 | -463.5 | 941.0 | 2416 | 191454 | 24640 | 51420 | 3.72E-04 | 4.82E-05 | Exp | Sud | 102.48 |
| 9 | -463.5 | 941.0 | 2377 | 194160 | 24715 | 51019 | 3.64E-04 | 4.87E-05 | Exp | Sud | 100.8 |
| 10 | -463.6 | 941.1 | 2875 | 183389 | 27661 | 50524 | 3.51E-04 | 4.47E-05 | Exp | Sud | 121.95 |
| 11 | -464.2 | 942.4 | 3965 | 161030 | 25026 | 52259 | 4.00E-04 | 3.49E-05 | Exp | Sud | 168.17 |
| 12 | -464.3 | 942.7 | 2784 | 186783 | 21775 | 52122 | 4.04E-04 | 4.74E-05 | Exp | Sud | 118.09 |
| 13 | -464.5 | 943.1 | 3683 | 164952 | 21879 | 53720 | 4.25E-04 | 3.83E-05 | Exp | Sud | 156.19 |
| 14 | -466.1 | 946.1 | 3320 | 182834 | 25711 | 51244 | 3.36E-04 | 5.16E-05 | Exp | Sud | 140.79 |
| 15 | -467.1 | 948.2 | 2548 | 196437 | 20164 | 50859 | 4.00E-04 | 4.67E-05 | Exp | Sud | 108.07 |
| 16 | -474.5 | 962.9 | 2830 | 199957 | 16785 | 66748 | 5.21E-04 | 1.77E-07 | Exp | Lin | 120.04 |
| 17 | -476.0 | 966.0 | 4137 | 201797 | 19561 | 53282 | 2.98E-04 | 9.64E-05 | Exp | Lin | 175.46 |
| 18 | -476.4 | 966.7 | 4138 | 152265 | 17843 | 52981 | 5.43E-04 | 1.21E-07 | Exp | Sud | 175.5 |
| 19 | -485.7 | 985.4 | 8120 | 206885 | 10953 | 68117 | 6.16E-04 | 3.38E-31 | Exp | Lin | 344.34 |
| 20 | -518.0 | 1050.0 | 2382 | 191461 | 39130 | 30167 | 2.10E-07 | 3.89E-04 | Sud | Sud | 101.05 |
| Princess Charlotte Bay (pop1) vs. Lihou East (pop2) | | | | | | | | | | | |
| Rank | Log Likelihood | AIC | N ancestral | t1 | nu11 | nu12 | m1_12 | m1_21 | expansion type pop1 | expansion type pop2 | theta |
| 1 | -459.2 | 932.4 | 2969 | 205894 | 83137 | 18013 | 3.35E-05 | 3.55E-04 | Lin | Exp | 125.93 |
| 2 | -459.5 | 933.1 | 2629 | 210277 | 84462 | 16305 | 3.53E-05 | 3.75E-04 | Lin | Exp | 111.48 |
| 3 | -460.5 | 935.0 | 3743 | 189455 | 86402 | 16677 | 2.77E-05 | 3.93E-04 | Lin | Exp | 158.75 |
| 4 | -462.1 | 938.1 | 3989 | 186364 | 88634 | 14533 | 2.81E-05 | 4.30E-04 | Lin | Exp | 169.16 |
| 5 | -470.0 | 954.0 | 3630 | 188666 | 86535 | 11678 | 1.38E-07 | 5.40E-04 | Lin | Exp | 153.96 |
| 6 | -470.3 | 954.7 | 4023 | 182952 | 87848 | 11517 | 1.24E-07 | 5.54E-04 | Lin | Exp | 170.6 |
| 7 | -470.4 | 954.9 | 4113 | 181793 | 87983 | 11627 | 1.22E-07 | 5.48E-04 | Lin | Exp | 174.43 |
| 8 | -470.9 | 955.7 | 3197 | 194605 | 86607 | 10488 | 1.56E-07 | 5.84E-04 | Lin | Exp | 135.57 |
| 9 | -471.4 | 956.8 | 2727 | 203462 | 83231 | 11628 | 1.83E-07 | 5.37E-04 | Lin | Exp | 115.64 |
| 10 | -472.0 | 958.1 | 2542 | 209701 | 75155 | 17604 | 8.84E-05 | 2.33E-04 | Lin | Exp | 107.8 |
| 11 | -473.1 | 960.2 | 2579 | 212651 | 73389 | 17986 | 9.63E-05 | 2.23E-04 | Lin | Exp | 109.4 |
| 12 | -474.2 | 962.4 | 4807 | 188399 | 75313 | 16724 | 9.02E-05 | 2.39E-04 | Lin | Exp | 203.87 |
| 13 | -475.2 | 964.4 | 5177 | 427121 | 108365 | 14631 | 6.57E-05 | 2.83E-04 | Exp | Exp | 219.56 |
| 14 | -475.5 | 965.0 | 7010 | 511991 | 108878 | 13665 | 6.31E-05 | 3.07E-04 | Exp | Exp | 297.25 |
| 15 | -475.6 | 965.1 | 7636 | 446241 | 108113 | 14616 | 7.02E-05 | 2.77E-04 | Exp | Lin | 323.81 |
| 16 | -475.9 | 965.8 | 9049 | 410653 | 106996 | 15862 | 7.33E-05 | 2.47E-04 | Exp | Exp | 383.71 |
| 17 | -476.4 | 966.9 | 4368 | 194136 | 79240 | 16498 | 1.18E-04 | 2.38E-04 | Lin | Exp | 185.26 |
| 18 | -481.5 | 977.0 | 7551 | 570121 | 121879 | 8059 | 6.62E-08 | 6.05E-04 | Exp | Exp | 320.21 |
| 19 | -527.6 | 1069.2 | 2444 | 201669 | 75948 | 36638 | 2.66E-04 | 2.05E-07 | Exp | Sud | 103.66 |
| 20 | -547.1 | 1108.2 | 11943 | 263827 | 59267 | 44558 | 2.49E-04 | 4.19E-08 | Exp | Lin | 506.44 |

AIC: Akaike information criterion; N ancestral: estimated ancestral population size before the split; t1: estimated time since divergence from the ancestral population; nu11: estimated contemporary effective population size of pop1; nu12: estimated contemporary effective population size of pop2; m1_12: estimated relative migration rate from pop2 to pop1; m1_21: estimated relative migration rate from pop1 to pop2. Lin: linear expansion; Exp: exponential expansion; Sud: sudden expansion.

**Table S3.** Summary of the top 20 ranked “complex” model comparisons with model structure set to one time interval before the population split and two time intervals after the split (1,2). Models are ordered by rank (1 to 20) according to AIC scores. Flinders Reef vs. Britomart Reef and Lihou East vs. Princess Charlotte Bay are shown in the top and bottom of the table, respectively.

| Flinders Reef (pop 1) vs. Britomart Reef (pop2) | | | | | | | | | | | | | | | |
| --- | --- | --- | --- | --- | --- | --- | --- | --- | --- | --- | --- | --- | --- | --- | --- |
| Rank | Log Likelihood | AIC | N ancestral | t1 | nu11 | nu12 | m1_12 | m1_21 | t2 | nu21 | nu22 | m2_12 | m2_21 | theta |  |
| 1 | -448.29 | 916.59 | 3659 | 159616 | 11657 | 40326 | 8.1E-05 | 6.9E-05 | 4772 | 32410 | 365650 | 9.1E-04 | 1.3E-05 | 155.2 |  |
| 2 | -448.3 | 916.59 | 3672 | 159407 | 11671 | 40410 | 8.2E-05 | 6.9E-05 | 4723 | 32180 | 367175 | 9.2E-04 | 9.9E-06 | 155.7 |  |
| 3 | -448.3 | 916.59 | 3678 | 159334 | 11699 | 40420 | 8.1E-05 | 6.9E-05 | 4715 | 32041 | 367700 | 9.2E-04 | 9.6E-06 | 156.0 |  |
| 4 | -448.3 | 916.59 | 3677 | 159467 | 11701 | 40410 | 8.1E-05 | 7.0E-05 | 4706 | 32059 | 367593 | 9.2E-04 | 8.9E-06 | 155.9 |  |
| 5 | -448.3 | 916.6 | 3689 | 159185 | 11743 | 40456 | 8.0E-05 | 6.9E-05 | 4658 | 32000 | 368936 | 9.3E-04 | 1.2E-05 | 156.5 |  |
| 6 | -448.57 | 917.14 | 3442 | 162884 | 10842 | 40558 | 1.1E-04 | 7.4E-05 | 5116 | 42138 | 254267 | 7.9E-04 | 1.6E-08 | 146.0 |  |
| 7 | -450.04 | 920.07 | 2863 | 171403 | 8018 | 41938 | 1.5E-04 | 7.5E-05 | 7751 | 37692 | 102494 | 5.9E-04 | 6.0E-08 | 121.4 |  |
| 8 | -450.2 | 920.4 | 3899 | 150833 | 5654 | 48369 | 2.0E-04 | 5.7E-05 | 8320 | 31323 | 65371 | 6.4E-04 | 6.2E-08 | 165.4 |  |
| 9 | -452.05 | 924.1 | 4005 | 147580 | 3378 | 50135 | 3.9E-04 | 6.9E-08 | 9987 | 36204 | 66099 | 5.0E-04 | 7.1E-05 | 169.8 |  |
| 10 | -452.15 | 924.29 | 3814 | 149675 | 2911 | 49147 | 4.6E-04 | 5.0E-05 | 10656 | 33868 | 68244 | 5.1E-04 | 1.1E-08 | 161.8 |  |
| 11 | -452.34 | 924.68 | 3953 | 147104 | 2646 | 49608 | 4.8E-04 | 4.5E-05 | 11258 | 32702 | 63939 | 5.1E-04 | 1.9E-10 | 167.7 |  |
| 12 | -453.69 | 927.38 | 3626 | 150901 | 1379 | 51017 | 1.0E-03 | 1.5E-09 | 12351 | 36686 | 60837 | 4.3E-04 | 1.8E-05 | 153.8 |  |
| 13 | -454.14 | 928.28 | 3828 | 148342 | 1206 | 51698 | 1.0E-03 | 1.3E-28 | 11250 | 38191 | 65129 | 4.7E-04 | 1.3E-07 | 162.4 |  |
| 14 | -454.17 | 928.34 | 3603 | 147256 | 1779 | 47714 | 3.5E-04 | 0.0E+00 | 16847 | 26652 | 61641 | 4.7E-04 | 4.7E-05 | 152.8 |  |
| 15 | -458.83 | 937.66 | 4252 | 150229 | 3470 | 50072 | 5.8E-04 | 1.3E-05 | 5128 | 58139 | 230194 | 7.5E-04 | 2.7E-08 | 180.3 |  |
| 16 | -473.58 | 967.15 | 2078 | 154434 | 670 | 45511 | 3.6E-04 | 1.2E-08 | 43224 | 18009 | 48489 | 3.1E-04 | 1.1E-04 | 88.1 |  |
| 17 | -476.32 | 972.63 | 2049 | 130306 | 2385 | 34512 | 7.5E-09 | 1.7E-06 | 61831 | 17252 | 47391 | 3.3E-04 | 1.0E-04 | 86.9 |  |
| 18 | -477.43 | 974.86 | 1531 | 126297 | 12988 | 11530 | 1.0E-03 | 1.0E-03 | 79187 | 16816 | 48709 | 3.3E-04 | 9.9E-05 | 64.9 |  |
| 19 | -477.49 | 974.97 | 1581 | 130442 | 9945 | 14628 | 5.0E-04 | 3.4E-04 | 78298 | 16853 | 48673 | 3.3E-04 | 9.9E-05 | 67.1 |  |
| 20 | -478.37 | 976.73 | 2789 | 102839 | 13501 | 14003 | 2.0E-03 | 2.0E-03 | 76521 | 15924 | 49477 | 3.6E-04 | 9.0E-05 | 118.3 |  |
| Princess Charlotte Bay (pop1) vs. Lihou East (pop2) | | | | | | | | | | | | | | | |
| Rank | Log Likelihood | AIC | N ancestral | t1 | nu11 | nu12 | m1_12 | m1_21 | t2 | nu21 | nu22 | m2_12 | m2_21 | theta |  |
| 1 | -448.68 | 917.35 | 3713 | 162997 | 46953 | 6144 | 6.7E-05 | 1.8E-04 | 12330 | 371385 | 22243 | 8.9E-09 | 3.8E-04 | 157.5 |  |
| 2 | -448.74 | 917.47 | 3844 | 160481 | 47212 | 5945 | 5.8E-05 | 1.6E-04 | 12521 | 384458 | 18963 | 0.0E+00 | 4.3E-04 | 163.0 |  |
| 3 | -448.85 | 917.7 | 3264 | 175730 | 46009 | 8505 | 7.5E-05 | 8.9E-05 | 11660 | 326428 | 17688 | 0.0E+00 | 4.5E-04 | 138.4 |  |
| 4 | -448.92 | 917.85 | 3714 | 164556 | 44014 | 7809 | 6.1E-05 | 9.1E-05 | 13482 | 371496 | 17345 | 7.7E-06 | 4.3E-04 | 157.5 |  |
| 5 | -448.94 | 917.89 | 3499 | 167273 | 46013 | 7183 | 7.7E-05 | 1.6E-04 | 12161 | 349993 | 23951 | 1.6E-09 | 3.5E-04 | 148.4 |  |
| 6 | -448.98 | 917.97 | 3589 | 165964 | 43565 | 7847 | 6.0E-05 | 8.5E-05 | 13837 | 358993 | 17023 | 1.0E-05 | 4.3E-04 | 152.2 |  |
| 7 | -448.99 | 917.98 | 2986 | 180156 | 46143 | 8264 | 8.5E-05 | 1.1E-04 | 11973 | 298683 | 20008 | 3.3E-09 | 4.0E-04 | 126.7 |  |
| 8 | -449.09 | 918.18 | 3513 | 166612 | 46728 | 8575 | 8.0E-05 | 1.2E-04 | 11230 | 351371 | 22121 | 2.1E-06 | 3.8E-04 | 149.0 |  |
| 9 | -449.13 | 918.25 | 3825 | 162425 | 43585 | 8966 | 6.5E-05 | 8.2E-05 | 13093 | 382553 | 17783 | 1.5E-05 | 4.1E-04 | 162.2 |  |
| 10 | -449.15 | 918.3 | 3124 | 177652 | 44931 | 8115 | 7.1E-05 | 8.7E-05 | 12740 | 311846 | 16544 | 7.3E-10 | 4.4E-04 | 132.5 |  |
| 11 | -449.25 | 918.51 | 4176 | 157656 | 49321 | 7318 | 6.4E-05 | 1.3E-04 | 10295 | 417607 | 18034 | 0.0E+00 | 4.8E-04 | 177.1 |  |
| 12 | -449.56 | 919.13 | 2897 | 177959 | 45149 | 6123 | 8.0E-05 | 1.8E-04 | 13890 | 289754 | 22614 | 0.0E+00 | 3.4E-04 | 122.9 |  |
| 13 | -449.92 | 919.85 | 3415 | 165796 | 46934 | 4122 | 5.2E-05 | 1.8E-04 | 15423 | 230746 | 17119 | 1.5E-08 | 4.3E-04 | 144.8 |  |
| 14 | -450.31 | 920.62 | 2309 | 190076 | 41827 | 7508 | 7.7E-05 | 9.3E-05 | 16051 | 230961 | 18631 | 6.0E-06 | 3.7E-04 | 97.9 |  |
| 15 | -451.6 | 923.21 | 3268 | 176860 | 38247 | 13932 | 7.2E-05 | 1.5E-07 | 14862 | 326817 | 13996 | 2.5E-05 | 4.6E-04 | 138.6 |  |
| 16 | -452.35 | 924.7 | 4463 | 149520 | 53643 | 2048 | 8.8E-07 | 9.1E-04 | 11638 | 398958 | 31302 | 7.5E-06 | 3.1E-04 | 189.3 |  |
| 17 | -452.36 | 924.71 | 3293 | 163938 | 50022 | 1543 | 6.3E-05 | 5.1E-04 | 19531 | 143836 | 16685 | 4.9E-08 | 4.0E-04 | 139.7 |  |
| 18 | -453.39 | 926.77 | 3859 | 158093 | 65436 | 1217 | 1.4E-04 | 1.0E-03 | 14201 | 121907 | 22520 | 0.0E+00 | 3.4E-04 | 163.7 |  |
| 19 | -454.68 | 929.35 | 4703 | 152347 | 44086 | 13343 | 5.9E-05 | 1.7E-20 | 11462 | 470339 | 11000 | 1.5E-05 | 6.4E-04 | 199.4 |  |
| 20 | -471.77 | 963.54 | 3453 | 126841 | 6638 | 36508 | 1.0E-03 | 2.8E-05 | 55218 | 83777 | 12909 | 5.8E-05 | 3.3E-04 | 146.5 |  |

AIC: Akaike information criterion; N ancestral: estimated ancestral population size before the split; t1: estimated time since divergence during the first time interval; nu11: estimated contemporary effective population size of pop1 during the first time interval; nu12: estimated contemporary effective population size of pop2 during the first time interval; m1_12: estimated relative migration rate from pop2 to pop1 during the first time interval; m1_21: estimated relative migration rate from pop1 to pop2 during the first time interval. t2: estimated time since divergence during the second time interval; nu21: estimated contemporary effective population size of pop1 during the second time interval; nu22: estimated contemporary effective population size of pop2 during the second time interval; m2_12: estimated relative migration rate from pop2 to pop1 during the second time interval; m2_21: estimated relative migration rate from pop1 to pop2 during the first time interval.

**Table S4**. Parameter settings used for marker filtering in Radiator v 1.2.1 (Gosselin, 2022) and subsequent number of loci retained or excluded at each step. The final number of loci retained for population genetic analyses are indicated in bold.

| Filter | Loci excluded | Loci remaining after filter |
| --- | --- | --- |
| All markers from Stacks | - | 17,474 |
| Filter monomorphic markers | 58 | 17,416 |
| Minor allele count > 4 | 9,650 | 7,766 |
| Coverage 10 X to 100 X | 442 | 7,324 |
| Missing genotype (> 0.1) | 2,432 | 4,892 |
| HWE 2 pops mid *p* < 0.05 | 83 | 4,809 |
| Heterozygosity (> 0.5) | 31 | 4,778 |
| Outlier loci (PCAdapt & Bayescan) | 230 | 4,548 |
| Total loci | - | **4,548** |

**Table S4.** Relative admixture proportions of *Plectropomus leopardus* individuals from the Coral Sea and the Great Barrier Reef (GBR) screened as mixed-ancestry versus reference samples of *P. laevis* and *P. maculatus*.

| Region | Reef | *Plectropomus leopardus* | *Plectropomus laevis* | *Plectropomus maculatus* |
| --- | --- | --- | --- | --- |
| Coral Sea | Flinders Reef | 0.97 | 0.03 | 0.00 |
| Coral Sea | East Lihou | 0.29 | 0.72 | 0.00 |
| Coral Sea | East Lihou | 0.52 | 0.48 | 0.00 |
| Coral Sea | East Lihou | 0.71 | 0.29 | 0.00 |
| Coral Sea | East Lihou | 0.80 | 0.20 | 0.00 |
| Coral Sea | East Lihou | 0.87 | 0.87 | 0.00 |
| Coral Sea | East Lihou | 0.90 | 0.10 | 0.00 |
| Coral Sea | East Lihou | 0.91 | 0.09 | 0.00 |
| Coral Sea | East Lihou | 0.97 | 0.03 | 0.00 |
| Coral Sea | East Lihou | 0.98 | 0.02 | 0.00 |
| Coral Sea | East Lihou | 0.98 | 0.02 | 0.00 |
| Coral Sea | West Lihou | 0.85 | 0.15 | 0.00 |
| Coral Sea | West Lihou | 0.87 | 0.13 | 0.00 |
| Coral Sea | West Lihou | 0.97 | 0.03 | 0.00 |
| GBR | Capricorn Bunkers | 0.84 | 0.01 | 0.16 |
| GBR | Capricorn Bunkers | 0.86 | 0.04 | 0.10 |
| GBR | Capricorn Bunkers | 0.88 | 0.00 | 0.12 |
| GBR | Capricorn Bunkers | 0.91 | 0.00 | 0.09 |
| GBR | Capricorn Bunkers | 0.93 | 0.00 | 0.05 |
| GBR | Princess Charlotte Bay | 0.84 | 0.00 | 0.17 |
| GBR | Princess Charlotte Bay | 0.90 | 0.09 | 0.01 |
| GBR | Swains Reefs | 0.49 | 0.51 | 0.00 |
| GBR | Swains Reefs | 0.49 | 0.51 | 0.00 |
| GBR | Swains Reefs | 0.51 | 0.50 | 0.00 |
| GBR | Swains Reefs | 0.91 | 0.00 | 0.09 |
| GBR | Swains Reefs | 0.95 | 0.03 | 0.01 |
| GBR | Torres Strait | 0.32 | 0.00 | 0.68 |
| GBR | Torres Strait | 0.75 | 0.00 | 0.25 |
| GBR | Britomart Reef | 0.96 | 0.00 | 0.04 |
| GBR | Britomart Reef | 0.98 | 0.00 | 0.01 |
| GBR | Britomart Reef | 0.98 | 0.00 | 0.02 |

***Supplemental figures***

**
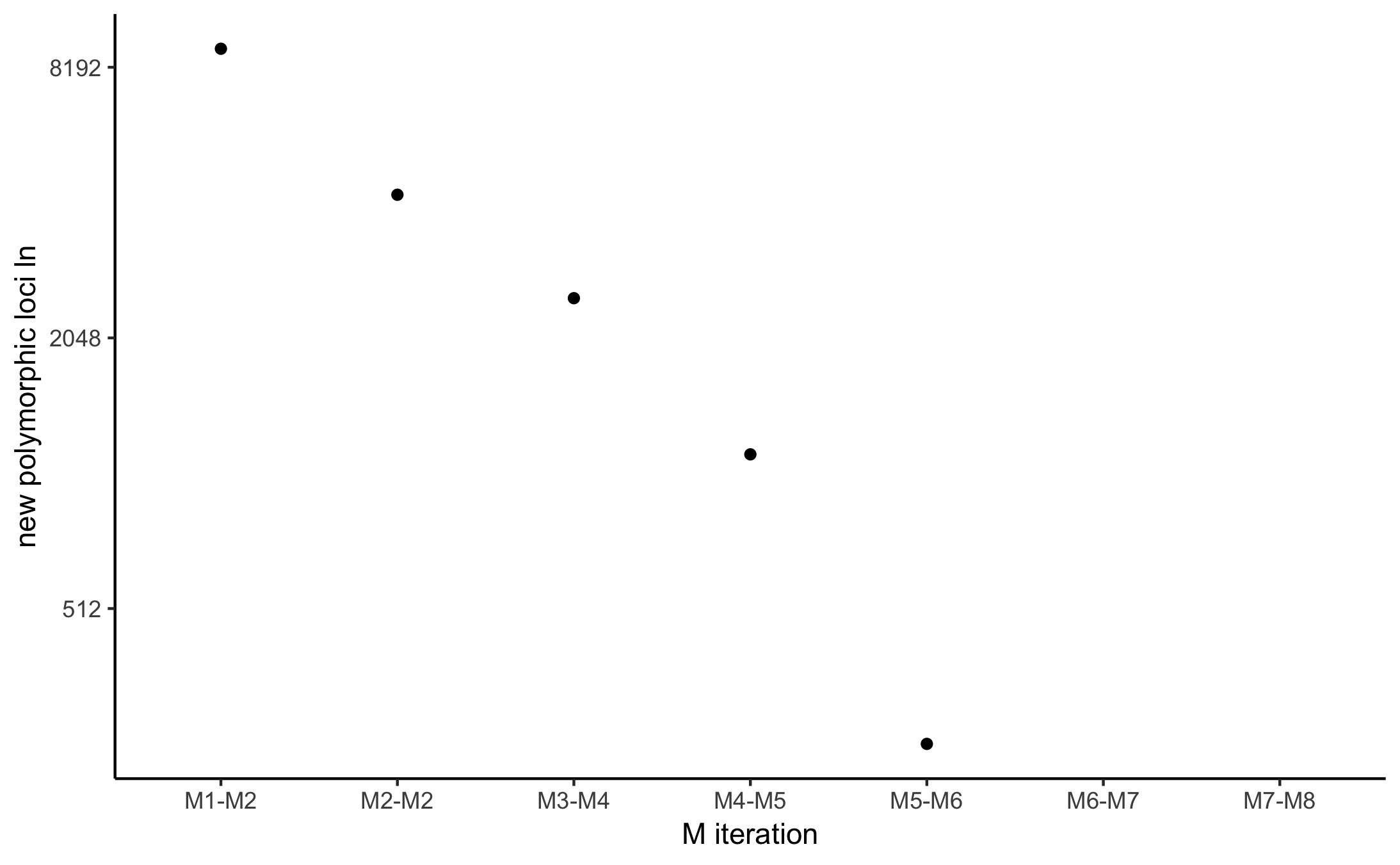
**

**Figure S1**. Total number of new polymorphic loci (log scale) for each increase in the parameter *M*.


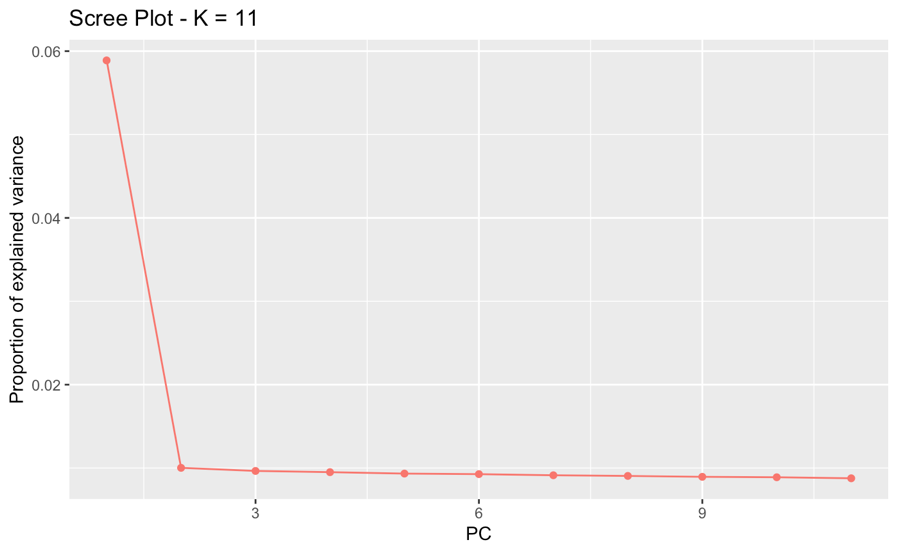


**Figure S2**. Screen plot results from PCAdapt implemented in the R package *Adegenet* (Jombart, 2008) indicating the most likely number of clusters (K) in our data.


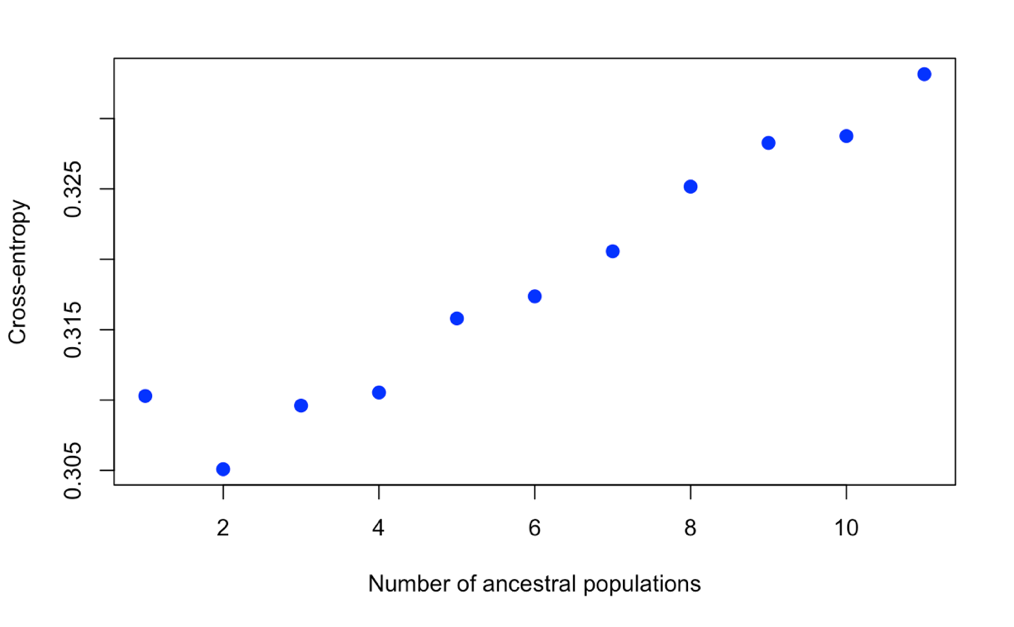


**Figure S3**. *K* cross entropy results from SNMF in the R Package *LEA* indicating the most likely number of genomic clusters identified in our data.

**Figure S4**. Pairwise *F_ST_* (Weir and Cockerham, 1984) heat map for *Plectropomus leopardus* collected in the Great Barrier Reef and the Coral Sea based on 230 outlier markers. Pairwise comparisons that were significantly different from zero at a corrected alpha threshold of 0.011 (Benjamini &Yekuteili, 2001) are indicated by a black circle.

**Figure S5**. a) Scatterplot of Discriminant Analyses of Principal Components (DAPC, *Adegenet* version 2.1.3, Jombart, 2008) for populations of *Plectropomus leopardus* sampled in the Great Barrier Reef (GBR) and Coral Sea based on 230 outlier markers. Bar plots of admixture coefficients for *P. leopardus* at b) *K* = 2 and c) *K* = 3 estimated using sparse nonnegative matrix factorization (SNMF) in the R package *LEA* (Frichot & François, 2014). Each vertical bar represents an individual and the colour indicates relative admixture coefficients at *K* ancestral populations. Populations are separated by black vertical lines and labelled according to the legend. GBR populations are ordered north to south from left to right, and Coral Sea populations are ordered by proximity to the GBR, from left (closest) to right (farthest).

**Figure S6**. Empirical and inferred joint site frequency spectra (top row) and residual plots (bottom row) for each replicate comparison (Flinders Reef versus Britomart Reef and East Lihou versus Princess Charlotte Bay). Inferred joint site frequency spectra were constructed using the diffusion approximation method (moments; Jouganous et al., 2017) implemented in the program GADMA (Noskova et al., 2020).

a)

b)

**Figure S7**. Best ranked “complex” demographic models describing divergence and changes in population size between a) Flinders Reef (Coral Sea) and Britomart Reef (Great Barrier Reef), and b) East Lihou (Coral Sea) and Princess Charlotte Bay (Great Barrier Reef). Demographic models were constructed using the diffusion approximation method (moments; Jouganous et al., 2017) implemented in the program GADMA (Noskova et al., 2020). Joint site frequency spectra for empirical and inferred data are shown in Figure S8.

**Figure S8**. Empirical and inferred joint site frequency spectra (top row) and residual plots (bottom row) for each replicate comparison (Flinders Reef versus Britomart Reef and East Lihou versus Princess Charlotte Bay). Inferred joint site frequency spectra were constructed using the diffusion approximation method (moments; Jouganous et al., 2017) implemented in the program GADMA (Noskova et al., 2020).

**References**

Benjamini, Y., & Yekutieli, D. (2001). The control of the false discovery rate in multiple testing under dependency. *Annals of Statistics*, 1165-1188.

Catchen, J., Hohenlohe, P. A., Bassham, S., Amores, A., & Cresko, W. A. (2013). Stacks: an analysis tool set for population genomics. *Molecular Ecology*, 22(11), 3124-3140.

Frichot, E., & François, O. (2015). LEA: An R package for landscape and ecological association studies. *Methods in Ecology and Evolution*, 6(8), 925-929.

Jombart, T. (2008). Adegenet: A R package for the multivariate analysis of genetic markers. *Bioinformatics*, 24, 1403–1405.

Jouganous, J., Long, W., Ragsdale, A. P., & Gravel, S. (2017). Inferring the joint demographic history of multiple populations: beyond the diffusion approximation. *Genetics*, 206(3), 1549-1567.

Noskova, E., Ulyantsev, V., Koepfli, K. P., O’Brien, S. J., & Dobrynin, P. (2020). GADMA: Genetic algorithm for inferring demographic history of multiple populations from allele frequency spectrum data. *GigaScience*, 9(3), giaa005.

Weir, B. S., & Cockerham, C. C. (1984). Estimating F-statistics for the analysis of population structure. *Evolution*, 1358-1370.

Gosselin, T. (2020). Radiator: RADseq Data Exploration, Manipulation and Visualization using R. R package version 1.2.1 https://thierrygosselin.github.io/radiator/.
